# Transactions of the American Medical Association

**Published:** 1850-05

**Authors:** 


### Part 2.—Reviews and Notices of New Works.

### ARTICLE I.

*Transactions of the American Medical Association, Instituted* 1847. Vol. 2 pp. 956, 8vo. Philadelphia: printed for the Association, by T. K. & P. G. Collins, 1848.

This anxiously expected volume has at length appeared on our table. The vexatious delay in its appearance was owing to the want of punctuality on the part of the various Committees, in furnishing their reports, and returning the proof sheets to the Publishing Committee. This statement is due to the Committee, in order to exonerate it from the charge of tardiness, which was everywhere being preferred against it. It is but common justice that the blame should attach to the proper persons.

The mechanical execution of the volume is excellent. We regret to see that a number of typographical errors of a vexatious character, have crept into the body of the work. We allude to the frequent misprint of the names of authors alluded to in the various reports. We suppose, however, that it is next to impossible to bring out such a volume, without mistakes of this character.

As this is, to the American Physician, the most interesting and important medical publication of the year, we shall not apologize to our readers for going somewhat into detail in our notice of its contents.

In the first part of the volume is contained the minutes of the session of the Association, an abstract of which we have already presented to our readers, in a preceding number.

The first report in the volume is that from the Committee on the Medical Sciences. To this report we have a few objections. The chairman of the Committee sets out by informing us that although instructed by the Association, to confine the report to the progress of the Medical Sciences in our own country, he had deemed it best to embody the discoveries in this department the world over. Now we have no doubt the entire profession concurs in the propriety of the instructions given by the Association to the Committees, and we do not recognize the right of the Committee to set at naught the specific directions laid down for its guidance. We are also informed by the chairman of the Committee that his files of American Medical Journals was found to be incomplete, and that this defect was not noticed until too late to remedy it. Of course this renders the report to some extent defective. We are told, however, in this report, that the Committee appointed for the present year, will probably be more careful in in this particular: and we hope the prediction will be verified. We are not informed whether or not the Committee's files of European Journals were incomplete; but we infer, from the abundant reference to them, that they were not.

We submit a brief synopsis of the more interesting points of this report, which have not heretofore been brought to the notice of our readers. Prof. Parker has revived the views of McIntosh, touching the pathology of Dysmenorrhœa, and gives the following result of Treatment.

"PATHOLOGY OF DYSMENORRHŒA.—Professor Parker has treated a number of cases of dysmenorrhœa within the past year, upon the theory that the difficulty lies in the narrowing of the os uteri, and perfect success has attended the practice. He relieves the stricture by introducing a bougie of sufficient size, precisely as he would remove ordinary strictures from the urethra. From much observation, he is satisfied that many, if not most, cases of dysmenorrhœa depend upon this mechanical obstruction, and that the mechanical treatment proposed will save numbers of the sex from years of intense suffering, and relieve them from the evil of sterility."

Dr. Cartwright is responsible for the following, in which, the rapid increase of Western population is accounted for, in the most natural and satisfactory manner in the world:

"CAUSES OF MORTALITY IN THE VALLEY OF THE MISSISSIPPI.—Dr. Cartwright refers the mortality of the States west of the mountains to three principal causes. 1st. Malaria, and the too common practice of treating malarious or bilious affections, congestive fever, pneumonia, &c., as the diseases of hyperborean Europe are treated. 2dly. The large amount of adulterated, stale, and worthless medicine brought into those States and used by physicians. 3dly. The immense consumption of nostrums and patent medicines of all kinds by the "western people in attempting to doctor themselves." The mortality of the Western States does not appear as conspicuously as it ought, "owing to the women in the Mississippi valley being twice as *prolific*, or nearly so, as the women east of the Alleghany mountains. For every 1000 women east of the mountains, between the years of 15 and 50, there are only on an average about 1200 children, or persons under 15 years of age; while, for every 1000 women between 15 and 50 west of the mountains, the average number of children or persons under 15 years of age, is 2200. Owing to this greater fecundity of western women, if there were no emigration from the east, and the mortality in the west was the same, the west would double its population in nearly half the time of the east." But it does not do this, allowing for what it gains and the east loses by removals, and the deficiency is occasioned by the greater mortality of the valley of the Mississippi."

On page 97 we find a description on the therapeutic properties of Quinine. Dr. Booth "concludes that the power of the medicine depends on an *anti-congestive* property, the result of a tonic action on the nervous system." He thinks periodicity is always to be met with Quinine, whether inflammation be present or not; and then, inconsistently enough, discourages its use in Remittent Fever. Dr. Shanks regards it as a sedative, and administers it in acute inflammations.—Dr. Mendenhall thinks that although Quinine has some properties peculiar to itself, it must be classed as a stimulant. Dr. Brickell relates a case of traumatic tetanus, treated by large doses of this drug. 30 grs. in 60 drops of laudanum, were administered twice in one day, and three times the following day, with entire relief; and this, after chloroform had failed to alleviate the spasms.

Under the head of Medical Jurisprudence, some interesting items of information are given. Calcined Magnesia is strongly recommended as an antidote to Arsenic---the remedy to be administered *pro re nata.* The Carbonates of Potash and Soda are recommended as antidotes to the Chloride of Zinc.

At the close of this report is a careful, well written summary of the present condition of our knowledge on the subject of Anæsthetics. In our opinion, this is the most valuable portion of the report; but as it is itself an abstract, and as many of its most interesting points have already been presented to our readers, we content ourselves with heartily subscribing to the sentiment with which the report concludes: "That finally, with all the drawbacks upon them, Anæsthetics form a most precious contribution to the resources of our art."

The next document in the volume is the report of the Committee on Practical Medicine. Of all the Committees, we should suppose this one had the most difficult duty to perform. To collect the history of the epidemics and endemics over so large an extent of country as ours, would require that the Committee should be composed of persons from all quarters of the country, and that each member should industriously co-operate with the chairman.

The author of this report (Dr. D. F. Condie), is well known to the profession as a gentleman of most extensive research, and indefatigable industry; and we may therefore rest assured that no labor and pains have beeen spared to render the report interesting and valuable.

The principal epidemics of the year ending May 1849, were Typhus or Ship Fever, Erysipelas, Dysentery, Cerebro-Spinal Meningitis, Yellow Fever and Cholera.

Appended to the Report, are, First, A Report on *The More Prevalent Diseases of Swedesboro, N. J., and its Neighborhood, during the year* 1848: By Dr. J. F. Garrison.

Second, *An Account of the Dysentery, as it prevailed in Cambridge, Mass., in the years* 1847 *and* 1848: By Dr. Morrill Wyman. Dr. W, Placed his main reliance on Opium; and his treatment seems to have been attended by very satisfactory results.

Third, *On Bilious Fever, as it prevails in the Eastern portion of New Jersey:* By Dr. J. Fithian. Dr. F. thinks that when the fever is high, the best mode of treatment, is to precede the use of purgatives and quinine, by bleeding.

Fourth, *On Erysipelas, as it prevailed in the Eastern portion of New Jersey; with some Remarks on Erysipelas of the Respiratory Mucous Membrane:* By Dr. J. Fithian. His mode of treatment presents nothing peculiar.

The next document in order is the excellent report of the Committee on Surgery. This is short, concise, and very much to the purpose. The first part is taken up with a summary of Anæsthesia in Surgical Practice.

Of the Gutta Percha as a surgical appliance, the Chairman speaks as follows:

"Very recently, if not within the last year, a new, and remarkable material has been furnished to the profession, for the mechanical treatment of fractures. The Gutta Percha, to which we allude, possesses a property by which, when immersed in hot water, it becomes as plastic as wet paper. On cooling, it instantly recovers its firmness, which is almost equal to that of horn, retaining whatever form may have been impressed upon it when warm.

"This property will, at once, suggest to any one who has employed the starched bandage, the usefulness of the article, in the formation of moulded splints. We have, however, searched the communications of our correspondents, as well as the journals, in vain, for any reports of its use by *American* surgeons. Another year may furnish interesting facts in relation to it. We have ourselves employed it with great satisfaction in fracture of the patella and of the leg. Prof. Wiltenberger, of Baltimore, is now employing this form of splint in treating a case of ununited fracture after resection. He informs me that it accomplishes the mechanical purpose with beautiful precision."

Prof. Pope has used with good results, in the treatment of fractures of children, splints formed by saturating strips of linen with Collodion. He regards such a splint as superior to the starch or dextrine bandage. Our readers will perhaps recollect that a great noise was made sometime since, concerning Jarvis' *Patent* Surgical Adjuster. The fact of the apparatus being patented, led us to infer that there was probably something wrong about it; and we see that it is herein condemned as a deficient and unsatisfactory instrument.---Prof. Smith presented to the Association, a new method for the treatment of dislocation of the shoulder, which we here furnish to our readers:

* * * * "Its peculiarity chiefly consists in the application of the counter-extension to the opposite wrist, extension being made from the wrist of the dislocated member. Steady traction from the two wrists, in the horizontal direction, will be observed immediately to erect the head, neck, and chest, and to restore the symmetry of the two sides of the body. This at once calls to our aid the action of numerous muscles. The object of counter-extension, is, of course, to fix the scapula of the injured side. Traction from the opposite side most effectually does this, first, by erecting the spine, which otherwise yields to the extension, and, more directly, by communicating support to it through the clavicles which in front fix the two scapulæ together, and behind through the muscles and tendons, which effect the same on the back. Support one scapula and you necessarily sustain the other.

We accomplish reduction, in many instances of dislocation into the axilla, by simple traction, for a few minutes, from the two wrists. In difficult cases we place the patient on a chair, pass a band (sheet or towel) over the top of the saapula and tie it beneath the chair. The knee of the surgeon is then placed in the axilla, and traction is made steadily from the two wrists, till the muscles are observed to yield, and the head to be disengaged. Then the surgeon directs the arm to be depressed, while at the same moment he urges his knee into the axilla by extending his foot. We have succeeded thus in cases of two months' standing, and where other methods in judicious hands have failed. Indeed, we now practice no other method."

Dr. Bigelow uses Gutta Percha for taking impressions of stricture of the Uretha:

* * * * "He employes it thus: A bougie of this material is oiled, and then its point is passed to and fro rapidly, in the flame of a candle, till the nail will indent it. If it be now quickly passed down to the stricture and urged against it, with a force equal to an ounce or two of weight, and then allowed to cool during three or four minutes, it will retain, when withdrawn, a perfect impression, thus furnishing to the operator information, which will give precision to the mechanical means subsequently resorted to."

The report on Obstetrics is somewhat incomplete. The Chairman being incapacitated by continued ill health, from discharging the duty imposed upon him, a brief report was furnished by another member. There are two points which we deem worthy of notice, and therefore present them. The first has reference to treatment of Occlusion of the Vagina:

"This state of parts, resulting most commonly from injuries suffered during parturition, but sometimes from acrid leucorrhœal discharge, and sometime existing congenitally, has fallen under the notice of most obstetricians. During the past year, a new operation has been performed by Dr. A. P. Hayne, of Charleston, S. C. The adhesions of the vaginal wall were broken up by the preserving use of compressed sponge, and at the end of ten weeks the canal became pervious, and the retained menstrual fluid, which had been collecting for more than a year, was discharged; she did perfectly well.—This operation, if hereafter found effectual, has obvious advantages; on the one hand, over the use of the knife, as avoiding the great danger of cutting into the bladder or rectum; and on the other, over the operation by laceration, practised by Amussat, as inflicting much less pain. and producing less local and constitutional irritation. It deserves to be borne in mind as an available means of relieving cases of great danger and difficulty."

The second is a new mode of managing Placenta Prævia.

"It is to the active and original mind of Prof. Simpson, of Edinburgh, that we owe the revival of a practice first suggested by Mr. Kinder Wood, of Manchester, who proposed to separate the placenta entirely from the uterine surface, as a means of checking hemorrhage in placenta prævia. This new practice is advocated on three distinct grounds.

"1st. The old plan, of rupturing the membranes and turning the child, has been, according to recorded experience, singularly unsuccessful, one in three of the mothers perishing, and the proportion of children saved being inconsiderable.

"2d. In not a few cases, the rigidity of the os and cervix uteri renders the operation of turning exceedingly difficult; indeed, almost impossible; and, when performed under such circumstances, it is nearly always and of necessity fatal.

"3d. The detachment of the placenta will, in the vast majority of cases, immediately and effectually check the hemorrhage."

We here close for the present our notice of this valuable and interesting volume. We will, in a future number, resume the subject; as we feel assured that our readers cannot but be gratified with a summary so purely the exponent of professional progress in the United States.

This volume may be obtained by remitting Three Dollars to Dr. Isaac Hays, Philadelphia. M.

